# Experimental investigation of CO_2_ uptake in CO_2_ hydrates formation with amino acids as kinetic promoters and its dissociation at high temperature

**DOI:** 10.1038/s41598-022-12538-1

**Published:** 2022-05-19

**Authors:** Shubhangi Srivastava, Ann Mary Kollemparembil, Viktoria Zettel, Timo Claßen, Bernhard Gatternig, Antonio Delgado, Bernd Hitzmann

**Affiliations:** 1 Department of Process Analytics and Cereal Science, University of Hohenheim, 70599 Stuttgart, Germany; 2 Institute of Fluid Mechanics (LSTME), FAU Erlangen-Nuremberg, Erlangen, Germany; 3 Process Engineering and Circular Economy, University of Applied Sciences Weihenstephan-Triesdorf, Triesdorf, Germany; 4 German Engineering Research and Development Center LSTME Busan, Busan, Republic of Korea

**Keywords:** Carbon capture and storage, Nutrition

## Abstract

The dissociation of CO_2_ gas hydrates (GH) with amino acid kinetic promoters and without promoters was studied at a high temperature of 90 °C for a period of 20 min to understand the percentage of CO_2_ gas and to select the best promoter that aids CO_2_ gas entrapment along with stability at a high temperature. The possibility of using four hydrophobic food grade amino acids, namely cysteine, valine, leucine, and methionine, and one surfactant, lecithin, as kinetic promoters for CO_2_ GH has been studied. The amino acids were added 0.5 g (wt%), and lecithin was added 5 g for the GH production. Furthermore, the amino acids leucine and methionine gave some positive results, therefore, these amino acids were carried further for the experimentation purpose in the production of CO_2_ GH. Also, a combinational use of these amino acids was studied to investigate the effect on % CO_2_ retention in comparison to the normal GH. From the results, it was observed that the stability of GH decreases with an increase in temperature, but the addition of promoters, especially leucine + methionine + lecithin increased the CO_2_ uptake during GH formation.

## Introduction

Gas hydrates (GH) are formed when water and low molecular weight gases are subjected to appropriate temperature and pressure conditions. Suitable sized guest molecules are caged in hydrogen-bonded water molecules without chemical reactions so as to stabilize the structure. The most common guest molecules used for the GH are ethane, methane, butane, propane, nitrogen, and carbon dioxide^1,2^.

Lower temperature, high pressure, the presence of guest molecules, and the appropriate quantity of water molecules are the essential prerequisites for hydrate production. The creation process is physical rather than chemical. It should be noted that the guest molecule freely spins within the water molecules cavities. The creation of gas hydrates is a crystallization process that includes nucleation and crystal growth processes, followed by a large accumulation phase^3,4^. The mass transfer of the gas to the hydrate's surface is critical during hydrate formation and may dominate the process. Furthermore, the exothermic heat of hydrate production might influence hydrate development^2^. Chemical and mechanical approaches are frequently used to enhance the development of gas hydrates. Mechanical approaches aim to increase the contact area and mass transfer between water and gas, while the chemical method is utilized to accelerate hydrate formation under milder conditions, increase gas absorption and improve hydrate selectivity^5–12^.

Surprisingly, GH promoters can reduce the energy requirements for hydrate formation^13,14^. Promoters are available as two kinds: thermodynamic promoters or kinetic promoters. The application of thermodynamic promoters can accelerate the pace of GH production by lowering the hydrate's equilibrium pressure, but some hydration cages will be occupied by these promoters, resulting in a considerably lower CO_2_ concentration^1^. The kinetic promoters, on the other hand, will not change the hydrate equilibrium conditions but will considerably boost the gas absorption rate during hydrate formation by speeding up the nucleation process^15,16^. Due to environmental concerns as a source of excellent kinetic hydration promoters, amino acids have been proposed^17^. The addition of GH kinetic promoters can either lower the pressure requirements or raise the temperature at which the hydrates are stable. This reduces the amount of energy necessary to pressurize or cool the targeted systems^1,13,14^^.^ Furthermore, the current interest in CO_2_ GH is not confined to carbon capture and sequestration; a paradigm change is needed to consider CO_2_ GH as a material to be employed in food industrial processes^18^.

Amino acids, which are fundamental components of the human diet and hence eco-friendly materials, have recently emerged as a very powerful class of promoters and, unlike surfactants, offer a clean mode of kinetic action, i.e., no foam generation. An amino acid is composed of a hydrogen (H) atom, an amino (NH_2_) group, a carboxyl (COOH) group, and a distinct R group (or side chain). They are classed as polar (hydrophilic) or nonpolar (hydrophobic: cysteine, leucine, methionine, valine) based on the qualities of their unique side chains. Amino acids have received a lot of attention recently because of their great physicochemical features, such as low volatility, low toxicity, and good oxidative stability^19^. Some amino acids, such as methionine, valine, and leucine, have a substantial CO_2_ gas storage capacity in the form of hydrates and quicker gas absorption. Therefore, such amino acids may be valuable material for GH applications^1^. The fundamental benefit of amino acids is that they are naturally innocuous and biodegradable, and they are not expected to cost any more than other synthetic compounds. The kinetic hydrate promoters are advantageous in the formation of GH as they do not occupy the water cages and aid in promoting gas intake. Furthermore, kinetic GH promoters are functional at even low doses of 100 ppm, and they function primarily by reducing the surface tension between the gas and liquid phases.

Much research has been conducted to accelerate the rate of hydrate formation using hydrate promoters, including the use of surfactants^20,21^. Surfactants are surface-active chemicals that have both hydrophobic and hydrophilic components. As a result, they can dissolve both polar and non-polar compounds, and these hydrophobic/ hydrophilic groups are the property deciding variables for each surfactant. Also, they have the ability to change the surface or interfacial tension and the contact angle between the phases, resulting in changes in surface charge and surface viscosity. Lecithin is zwitterion surfactant made up of both cationic and anionic centres that are connected to the same molecule. The choice of an appropriate amino acid is crucial in CO_2_ absorption since the process's effectiveness is highly reliant on the solvent's behaviour^22^. A better solvent for CO_2_ capture should have a quicker absorption rate since it leads to shorter column diameters, lowering the absorber's capital cost^23^. Liu et al.^24^ was the first to investigate amino acids in CH_4_ GH promoters at low concentrations of upto 1% wt. At 0.5 wt percent, leucine promoted the most CH_4_ hydrates compared to phenylalanine, glutamic acid, arginine, tryptophan, methionine, and histidine^24,25^. The behaviour of amino acids can change depending upon the GH being studied. For example, histidine works as a kinetic promoter for CH_4_ GH^26^, but acts as a kinetic inhibitor for CO_2_ GH, suggesting that the kinetic promotion/inhibition impact of amino acids varies with the type of guest molecule present^27^. Interestingly, leucine and methionine can promote both CH_4_ and CO_2_ GH^1^.

In the current study, the CO_2_ GH is kept in focus, so to achieve a sustainable CO_2_ percentage in the GH for the application as a leavening agent in the food industry, specifically during the baking process, a clear understanding of the CO_2_ gas trapped in GH at high temperature is essential. The temperature stability of GH is important while baking due to the exposure of high temperature during various steps involved. In order to effectively use GH in the baking industry, good knowledge of the formation of GH, its gas containment capacity, and the effect on its properties by the addition of promoters is very useful. To be economically feasible, hydrate-based technological applications nearly usually require fast hydrate formation as well as strong gas uptake. The injection of certain additives into the system is one strategy that might be used to attain the same result. These additives are referred to as hydration promoters. The idea of employing hydrophobic amino acids as kinetic promoters for CO_2_ GH has been investigated in this study. Based on the literature survey, various amino acid promoters such as cysteine, valine, leucine, methionine, and a surfactant lecithin were studied to identify the temperature stability and gas entrapment of the produced CO_2_ GH at high temperature of 90 °C. Furthermore, because none of the previous research compared the performance of amino acids, the current study is meant to determine the best one among four distinct types of amino acids, and therefore, combinational use of amino acids (leucine and methionine) was studied to investigate the effect on percentage CO_2_ retention in comparison to the normal GH. In addition, lecithin was successfully employed as a surfactant in a hydrate reactor to disperse either water in the gas phase or gas in the water phase.

## Results

### Economic viability and optimization of the amount of amino acids being used as kinetic promoters for the CO_2_ GH production

To check the economic viability of the application of amino acids (leucine and methionine) as promoters for the CO_2_ GH, some calculations were done based on the molecular weight of the amino acids (Table 1). The number of moles consumed in each cycle was calculated by dividing the weight of the amino acid taken for the GH production by 1 mol per molecular weight of the respective amino acids. So, from the calculations, it was found that leucine and methionine consume only 0.003 g per mole in each cycle of the CO_2_ GH production (Table 1). Hence, the application of amino acids being used as kinetic promoters was quite economically feasible.

**Table 1:** Number of moles consumed for leucine and methionine in production of GH.

		Leucine as promoter	Methionine as promoter
1	Molecular Weight (g/ mol)	131.17	149.21
2	Amount taken for GH production (g)	0.5	0.5
3	No. of moles consumed in each cycle of GH production	0.003	0.003

Table 2 shows the effect of increasing the amount of amino acids on the percentage of CO_2_ present in the GH. Also, to optimise the amount of amino acids to be used in each cycle of production, an experiment was performed by calculating the percentage of CO_2_ present in the GH after 1 h at room temperature (Table 2). In this method, CO_2_ GH was kept on a weighing scale at room temperature to see its dissolution. Amino acids leucine and methionine when used in a gradient of 0.2 and 0.3% were not able to capture sufficient amount of CO_2_, but at a gradient of 0.5% both the amino acids captured sufficient amount of CO_2_ (Table 2). Therefore, it was found that an optimum amount % by weight of leucine or methionine contributed to higher CO_2_ entrapment at room temperature. Therefore, increasing the amount of amino acids to 1% did not significantly increase the proportion of the CO_2_ gas present in the GH, so it was further confirmed that 0.5% of amino acids, either be leucine or methionine were found to be the optimised levels for the production of the GH. So, the higher percentages of either leucine or methionine were not in favour of more uptake of CO_2_. As a result, the presence of amino acids beyond a specific threshold causes them to crystallize among themselves and very weakly interact with hydrate water^28,29^. Thus, the ability to form hydrates at higher mole fractions decreases^30–32^. Thereby, an optimum amount is required for the proper functioning of the amino acid as kinetic promoter in the CO_2_ GH which was then fixed to 0.5 g on a weight basis.

**Table 2:** Effect of increasing amount of amino acids to percentage of CO_2_ present in GH.

		0.2% leucine	0.3% leucine	0.5% leucine	1% leucine	0.2% methionine	0.3% methionine	0.5% methionine	1% methionine
1	Weight of GH in the beginning (g)	18.6	18.2	16.9	18.0	20.0	20.0	20.0	20.0
2	Weight of GH after one hour (g)	17.8	16.0	13.5	15.0	19.8	18.6	16.0	17.1
3	Percentage of water (%)	95.6	87.9	79.5	83.4	98.6	92.8	80.1	85.5
4	Percentage of CO_2_ (%)	4.3	12.0	20.4	16.5	1.2	7.0	19.9	14.5

### Comparative evaluation of GH produced with amino acids as kinetic promoters and without promoters

**Figure 1:**
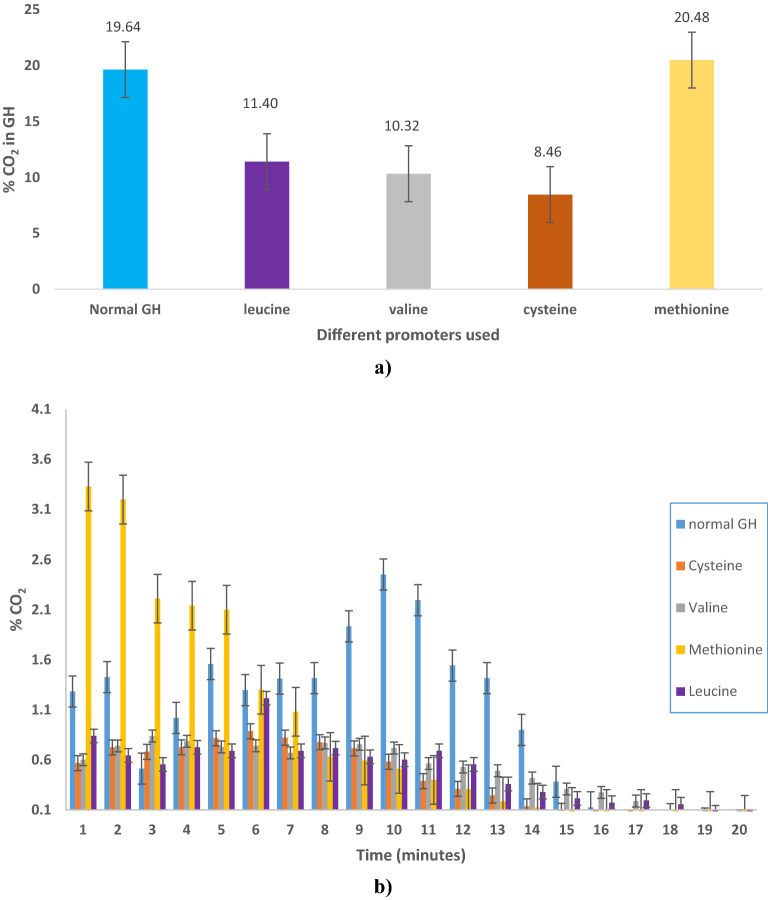
Comparative evaluation of percentage CO_2_ gas release (**a**) total, and (**b**) in parts with time from GH with amino acids as promoters.

In order to effectively use CO_2_ GH as a leavening agent in the baking industry, a concise evaluation of the formation of CO_2_ GH, and its gas containment capacity should be adequately analysed and documented. Also, the effect on CO_2_ GH properties by the addition of promoters should be taken into consideration as baking involves high temperature, and stabilising the GH at high temperature is an important criterion in the context of baking different products. Hence, the effect of the higher temperature of 90 °C was studied on the CO_2_ gas entrapment of the produced GH with kinetic promoters. Figure 1a shows the comparative evaluation of the total percentage CO_2_ gas release form the GH with and without promoters. The normal GH had a 19.64% CO_2_ gas release, while leucine had 11.40%, valine 10.32%, cysteine 8.46%, and methionine 20.48% of CO_2_ gas respectively. Figure 1b shows the comparative evaluation of the percentage CO_2_ gas release in parts with time from the GH without promoters (normal water GH) and amino acids (leucine, methionine, cysteine, and valine) as kinetic promoters. All of these experiments were carried out under the same experimental conditions, namely 0.5 wt % amino acids, a similar temperature range, initial gas pressure, and uniform cooling/warming rates because these parameters could have a significant impact on GH nucleation and gas uptake rate. The amino acids employed in this study have varying functional groups and hydrophobicity. Amino acids, used in the present study, possess different functional groups and hydrophobicity. Valine, cysteine, leucine, and methionine have aliphatic side chains, but methionine and cysteine has a sulphur atom in its side chain^33^. Also, it may be taken into consideration that temperature plays a major role in baking as well as in the disintegration of the GH with an increase in temperature. Although GH can be readily stable at lower temperatures, but utilising the CO_2_ GH to be functional as a leavening agent in the baking process is a challenge, thus a study focusing on the behaviour of the GH with the addition of amino acids as kinetic promoters was an important part. This was done to check if there is any difference when the CO_2_ GH are exposed to high temperature of baking.

At a temperature of 90 ºC, the maximum percentage of CO_2_ gas released with normal water GH was 2.45% in 10 min, and after that, it began to decrease. The GH prepared from leucine had a maximum CO_2_ release of 1.21% in 6 min, while the one prepared with methionine had a maximum CO_2_ release of 3.33% in 1 min. The GH prepared with the addition of cysteine and valine had a maximum CO_2_ release of 0.89% in 6 min, and 0.77% in 8 min, respectively. Moreover, the GH prepared with cysteine and valine did not gave a significant increase in the CO_2_ content in the GH with time in comparison to the ones that were prepared with normal water only. Also, the GH prepared with cysteine and valine were not consistent in nature and stability. The maximum gas release for all the amino acids was found in the time period of 1 to 7 min, and after that, it was just over. It was also discovered that the rate of dissociation diminishes steadily with time. This is because all of the trials were carried out under constant conditions. Furthermore, when the barrier to the transfer of CO_2_ gas from the gas to the liquid phase increases, the total mass-transfer coefficient steadily declines, suggesting GH dissociation^34,35^.

Although leucine had less amount of CO_2_ than the normal GH, but it was found that the GH prepared with leucine had more stability than the normal GH prepared with water alone. Moreover, the higher content of CO_2_ in the methionine application as a promoter in comparison to normal water prepared GH led to the selection of methionine as the best promoter. The basic explanation of why the amino acids leucine and methionine acted as good promoters in the case of CO_2_ GH is that both the amino acids are moderately hydrophobic in nature, and thus might prevent hydrate film formation at the gas–liquid interface^26,31,36–38^. Also, when the CO_2_ gas is dispersed in water, empty cavities may be left behind, forming a porous structure. Thus, an increase in the kinetics of hydrate formation was most probably due to the mass transfer rates of CO_2_. If this was not so, then it would have otherwise inhibited further gas diffusion and hydrate growth. Also, the influence of the high temperature of 90 °C really affected the percentages of CO_2_ in the GH. Therefore, leucine and methionine were found to be the best kinetic promoters when used individually in the production of the CO_2_ GH. The side-chain length and hydropathy index of amino acids are two more parameters that contribute to their promotion/inhibition actions^24^. The promoter effect has been linked to side chain length and components, hydrophobicity, and concentration of the promoter in water in a number of articles from different researchers^25,26^. In general, there is no unanimity, and the highlighted process is more enigmatic. All amino acids have an optimal concentration for each gas system, over which their promotion/inhibition influence is reduced. The optimal promoting impact of leucine in CH_4_ hydrate, for example, is between 0.3 and 0.5 wt percent^18^.

The process of amino acid hydrate promotion is governed by a number of parameters that are not well understood^24^. The proposed amino acid kinetic GH enhancement effect is thought to be caused by their surface activity and adsorption behaviour via capillary action^1,24^. The presence of amine and carboxylic acid groups, as well as a side chain, results in the molecular structure of most amino acids being both hydrophilic and hydrophobic. Furthermore, the side chain of an amino acid might change depending on its structure, charge, and polarity. As a result, they are amphiphilic molecules and can operate as surfactants. Because of their surfactant properties, such amino acids can limit the development and agglomeration of hydrate nucleus crystalline films at the gas/liquid interface, allowing more gas to dissolve in the liquid phase for high hydrate gas absorption. Kumar et al.^40^, Veluswamy et al.^25^, and Stern et al.^39^ found similar findings and reported that due to hydrophilic nitrile and carboxylic groups present in an amino acid molecule, it would attract CO_2_ gas molecules to itself, resulting in gas density enrichment in its vicinity, preventing GH particles from agglomerating and producing a stiff hydrate layer at the liquid–gas interface, which would obstruct further hydrate formation^25,39–42^. Thus, the GH formed with amino acids as kinetic promoters is extremely flexible and porous in nature, which accounts for their hydration promoting impact^25^. The presence of porous and flexible hydrates enhances surface adsorption at the gas/liquid interface. This allows more liquids to be drawn to the gas/liquid interface due to greater capillary action, resulting in high gas absorption into hydrate formation. It is critical to emphasize that the interplay between CO_2_ molecules and amino acids influences the amino acid promotion/inhibition process in CO_2_ systems^25,31,32,38^. The physical and chemical characteristics of amino acids are highly influenced by the side chain. The disturbance of the local water structure is a significant concern when exploring hydrophobic amino acids as kinetic promoters. It has been established that the hydrogen bond network between water molecules surrounding hydrophilic moieties of hydrophobic amino acids has been broken, whereas that around hydrophobic alkyl chains has been strengthened^36^. The degree to which these disturbances occur is determined by the hydrophobicity of the amino acids^37^. The degree of hydrophobicity of the amino acids varies significantly. It has previously been proven that amino acids with increased hydrophobicity are superior kinetic promoters for accelerating hydrate nucleation and development^38^. However, the results reported in this study did not match up with the hydrophobicity indices as a basis for the classification of the best kinetic amino acid promoters (the hydrophobicity of leucine is 3.8, valine is 4.2, cysteine is 2.5, and methionine is 1.9). The hydrophobicity of the amino acids employed in this investigation decreases as follows: valine > leucine > cysteine > methionine. While methionine and other amino acids are hydrophobic, the hydropathy indices show that they are weakly to moderately hydrophobic^31,38^. Given that amino acids with high hydrophobicity (valine and leucine) have been shown to significantly promote CH_4_ GH formation but only exhibit weak or no kinetic promotion activity in CO_2_ GH systems, we wonder if an amino acid's hydrophobic strength has any bearing on its kinetic promotion performance/preferences^43^. As a result, the current findings show that there is no relationship between hydrophobicity and hydrate production. Amino acids have previously been demonstrated to disrupt the local environment of water molecules and to be integrated into a portion of the hydrate crystal lattice via hydrogen bonding^31^. The amino acids, on the other hand, did not occupy the hydrate cages. The unusual inclusion of amino acids in the hydrate lattice is perplexing. Sa et al.^31^ reached this result based on lattice distortions and expansion found in CO_2_ GH, including glycine, valine, and alanine^31^.

Therefore, based on our results, one of the probable reasons for the mild/weak hydrophobic (methionine) amino acids performance can be related to the combination of amino acids with water, which creates a hydrophobic zone in the solution. In the context of CO_2_ GH formation, it is conceivable to imagine that a system containing strong hydrophobic amino acid in solution would hinder the inner polar CO_2_ molecules from assembling in its proximity due to the amino acid's significant non-polarity. The lack of gas-enriched zones in the solution surrounding the amino acid hydrophobic zones is likely to have a negative impact on the amino acids kinetic promotion activity, resulting in the weak kinetic enhancement observed for CO_2_ GH in the case of valine and cysteine^38,43–45^. In contrast, in the case of a system including mildly hydrophobic amino acid, the amphiphilic capabilities of the amino acid molecule come into play. In such a case, despite its low hydrophobicity, an amino acid molecule in solution would draw CO_2_ gas molecules to itself due to its hydrophilic nitrile and carboxylic groups, resulting in gas density enrichment in its proximity^27,46^. Also, when combined with the pre-existing local water ordering in the amino acid molecule's surroundings, the higher gas density leads to rapid GH nucleation and growth, and thus the kinetic enhancement of CO_2_ GH formation is seen in weakly hydrophobic amino acids like methionine^1,43,47^.

### Comparative evaluation of CO_2_ GH with combinational use of amino acids as kinetic promoters with respect to normal water CO_2_ GH

**Figure 2:**
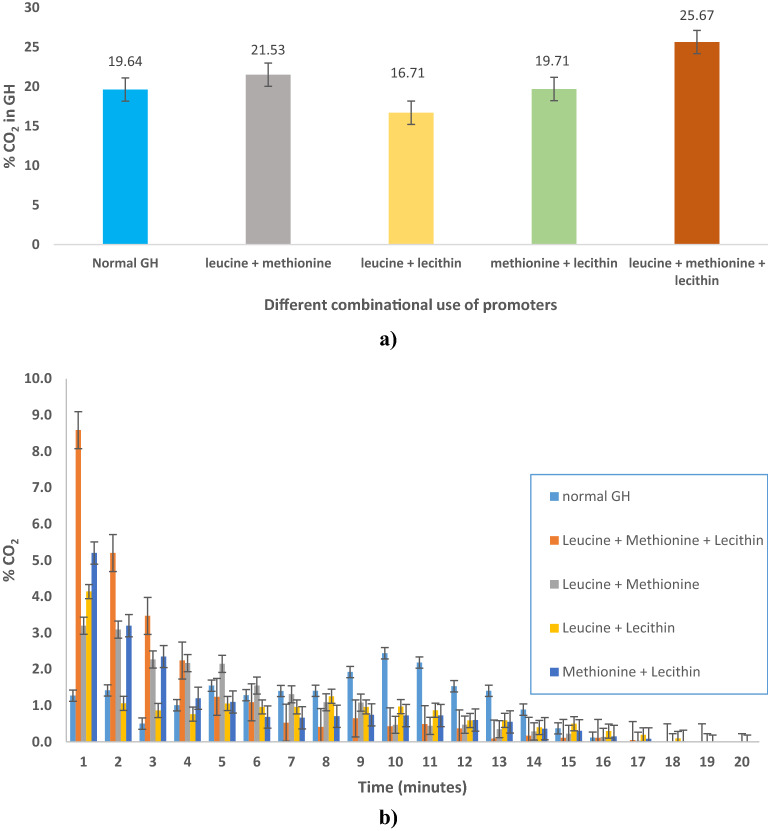
Comparative evaluation of percentage CO_2_ gas release (**a**) total, and (**b**) in parts with time from combinational use of amino acids promoters for GH.

**Figure 3:**
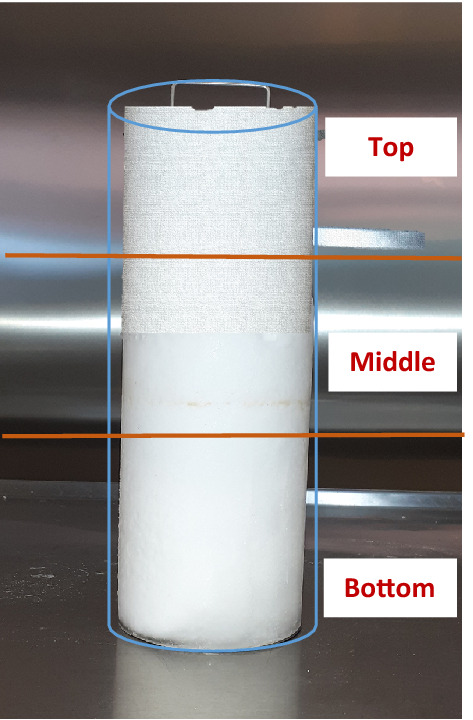
Diagrammatic illustration of division of different zones in a GH block prepared with promoters.

**Figure 4:**
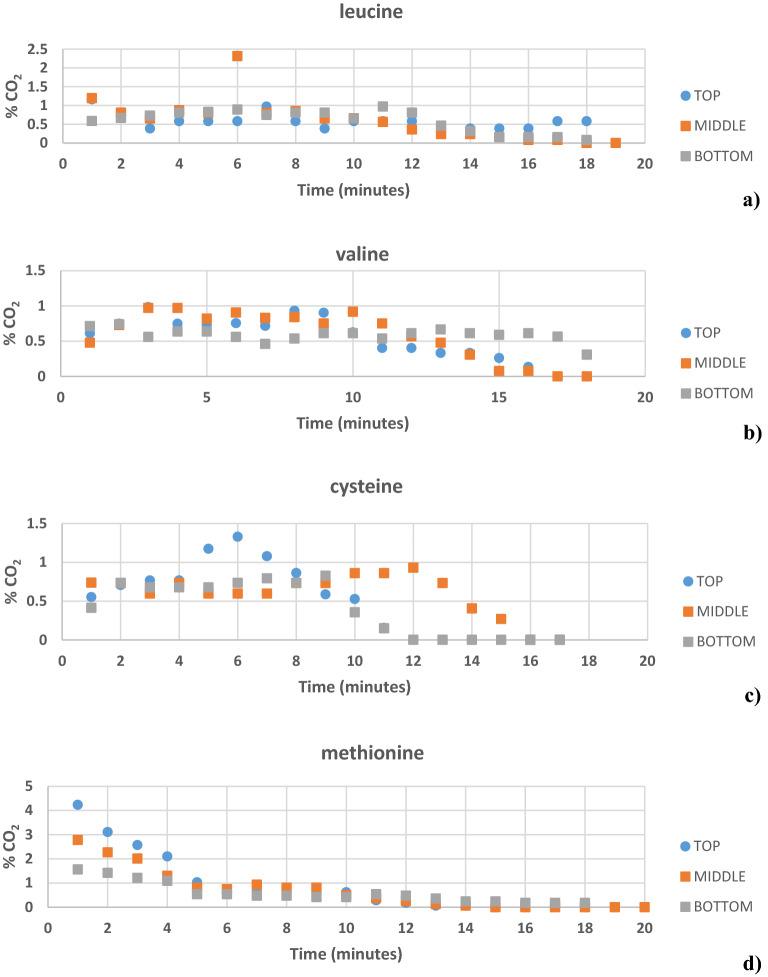
The % CO_2_ present in different zones of GH block with (**a**) leucine, (**b**) valine, (**c**) cysteine, and (**d**) methionine as promoter.

Figure 2a shows a comparative evaluation of the total percentage CO_2_ gas released from the combinational use of amino acids as kinetic promoters for GH. The normal GH had a CO_2_ gas release of 19.64%, while leucine + methionine had 21.53%, leucine + lecithin 16.71%, methionine + lecithin 19.71%, and leucine + methionine + lecithin 25.67% respectively. Figure 2b shows a comparative evaluation of the percentage CO_2_ gas released in parts with time from the combinational use of amino acids as kinetic promoters for GH. The GH prepared from the combination of methionine + lecithin had a maximum CO_2_ release of 5.03% in 1 min, while with leucine + lecithin combination had a CO_2_ release of 4.15% in 1 min, and the one prepared with leucine + methionine combination had a maximum CO_2_ release of 3.21% in 1 min, respectively. The GH prepared from the combinational use of leucine + methionine + lecithin had a maximum CO_2_ release of 8.59% in 1 min. Moreover, the gas uptake was higher in the lecithin + leucine + methionine system than in the leucine + lecithin, methionine + lecithin, and leucine + methionine in comparison to the normal water system. When compared to GH made from plain water, the addition of promoters increases the CO_2_ gas holding capacity. The best CO_2_ GH were the ones produced by a combination of lecithin, leucine, and methionine. Another phenomenon that can be reported from Figs. 3 and 4 is that the dissociation of the hydrates is not in a continuous decreasing trend. This discovery might be explained by a phenomena known as self-preservation. It has been shown that during GH dissociation, an ice layer forms around hydrate crystals, slowing down the GH dissociation rate even further^48,49^. Furthermore, at high temperatures, water molecules, and amino acid salts tend to migrate apart, causing hydrogen bonding to decrease. As a result, surface tension falls with temperature, which can aid in the selection of an absorbent with good surface tension performance (methionine) at high temperatures for the gas absorption process^29,50,51^.

These findings suggest that a mixture of amino acids aids CO_2_ molecules in occupying the GH cages more quickly. As expected from the results, not every amino acid can aid in the creation of CO_2_ GH equally, but the use of lecithin as a surfactant along with amino acids worked well. Moreover, it was proposed that the presence of surfactants causes the creation of micelles such as lecithin, which could enhance the solubility of the CO_2_ gas as well as act as nucleating sites^21,52^. The use of lecithin as a surfactant increases the solubility of a gas in water, which promotes the development of finer GH particles. These smaller GH particles have a larger surface area than the basic CO_2_ water system; as the surface area increases, so does the mass-transfer rate, resulting in a faster rate of production^2,35,53^. This suggests that by altering the development process of GH particles, lecithin solutions were able to convert more water to GH, and this provided a larger interface area during the GH formation^5,54–56^.

Despite decades of investigation, the process underlying GH production remains unknown. In this section, we offer a preliminary description of the promotion method of methionine + leucine + lecithin in the formation of CO_2_ GH from the structural aspect. Under CO_2_ GH formation circumstances, the amino acids act as zwitterions. These amino acids' electric charges allows them to connect with water molecules via strong electrostatic interactions. Furthermore, it has been shown that water molecules near electric charges become less "icelike"^57^. Secondarily, because of their amine and carboxylic acid groups, the amino acids employed in this work are more hydrophobic, but they also have a hydrophilic quality. As a result, they may interact with water molecules via strong hydrogen bonds^1^. Moreover, in leucine, the S atom in methionine is replaced by a C atom, the S atom is not the primary reason for methionine's great promoting function in the creation of CO_2_. An appropriate hydrophobic chain length in hydrophobic amino acids can enhance CO_2_ GH creation, and methionine stems from a suitable hydrophobic chain length, and the synergistic impact of hydrophilic carboxyl and amino groups also favours it as a good promoter of CO_2_ GH formation^29^. It can also be speculated that methionine, as an amphiphilic molecule, may function as a dispersion, preventing GH particles from agglomerating and producing a stiff hydrate layer at the liquid–gas interface, which would obstruct further hydrate formation^39,40^.

Moreover, referring to lecithin to be able to behave synergistically with the amino acids methionine and leucine in the formation of the CO_2_ GH comes from the fact that the choline and phosphate groups of lecithin molecules are attracted to neighbouring lecithin molecules when they are adsorbed onto the CO_2_ GH surface. Lecithin is a highly powerful hydration stabilizer (surfactant/promoter)^58,59^. Initially, because of the presence of lecithin molecules at the onset of the dissociation at high temperatures, the liberated water, and CO_2_ molecules had less moving space, resulting in slower dissociation. However, the discrepancies in dissociation rates with various amino acids may be attributable to the two lecithin layers on the GH surface, which attract water molecules dissociated from the GH, lowering the amount of space available for movement for the hydrate water and CO_2_ molecules. Thus, two amino acids (leucine and methionine) along with the surfactant lecithin result in higher CO_2_ capture through their synergistic effect as kinetic promoters for the CO_2_ GH.

Therefore, it is imaginable that surfactants and hydrophobic amino acids would influence CO_2_ GH formation either by reducing the time required for GH nucleation and/or increasing the rate of GH development. The aforementioned results determines that amino acids can be employed as possible blended kinetic promoters for CO_2_ GH production, somewhat better than separate and commercially available choices. This raises the prospect that combining leucine and methionine amino acids might boost their effectiveness by blending with surfactants such as lecithin. The results also showed that, as compared to pure water, the inclusion of these amino acids as kinetic promoters boosted gas absorption and water-to-hydrate conversion. However, the dissociation behaviour is much more sensitive to an increase in temperature.

### Comparison of different zones in a GH block prepared with promoters

**Figure 5:**
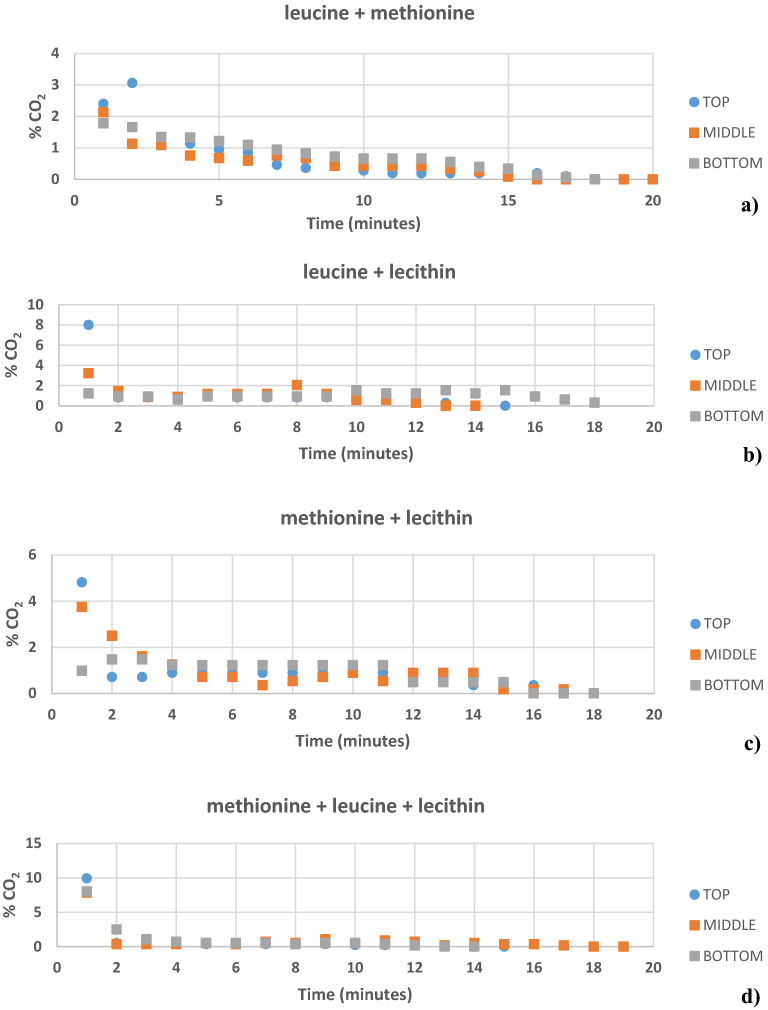
The % CO_2_ present in different zones of GH block with (**a**) leucine + methionine, (**b**) leucine + lecithin, (**c**) methionine + lecithin, and (**d**) methionine + leucine + lecithin as promoter.

Figure 3 shows the diagrammatic illustration of the division of different zones in a GH block prepared with amino acids as kinetic promoters. A comparison of the % CO_2_ in GH from the block layers (top/middle/bottom) was done for the GH prepared with amino acids as kinetic promoters the figures was plotted by taking average of three repetitions. This was done to check if the GH had an equal amount of GH % or if it differed in the three zones during the hydrate formation. Figure 4 (a-d) shows the % of CO_2_ present in different zones of the GH block with leucine, valine, cysteine, and methionine as amino acids as kinetic promoters for the CO_2_ GH, respectively. The maximum leucine % of CO_2_ was 2.31 at 6 min in the middle layer of the GH block, while in the top and bottom layers the maximum % of CO_2_ was 1.19 and 0.97 at 1 min, respectively. Overall, the middle layer captured more CO_2_, followed by the bottom and top layers. In case of valine, the maximum % of CO_2_ was 0.97 at 2 min in the middle layer of the GH block, while in the top and bottom layers the maximum % of CO_2_ was 0.93 (8 min) and 0.74 (4 min) respectively. Overall, the middle layer captured more CO_2_, followed by the top and bottom layers. In case of cysteine, the maximum % of CO_2_ was 1.33 at 6 min in the top layer of the GH block, while in the middle and bottom layers the maximum % of CO_2_ was 0.93 (12 min) and 0.83 (9 min) respectively. Overall, the top layer captured more CO_2_, followed by the middle and bottom layers. In case of methionine, the maximum % of CO_2_ was 4.24 at 1 min in the top layer of the GH block, while in the middle and bottom layers the maximum % of CO_2_ was 2.78 (1 min) and 1.56 (1 min) respectively. Overall, the top layer captured more CO_2_, followed by the middle and bottom layers. Figure 5(a-d) shows the % of CO_2_ present in different zones of the GH block with leucine + methionine, leucine + lecithin, methionine + lecithin, and methionine + leucine + lecithin amino acids/surfactants as kinetic promoters of the CO_2_ GH, respectively. In case of leucine + methionine, the maximum % of CO_2_ was 3.06 at 2 min in the top layer of the GH block, while in the middle and bottom layers the maximum % of CO_2_ was 2.13 (1 min) and 1.78 (1 min) respectively. Overall, the top layer captured more CO_2_, followed by the middle and bottom layers. In case of leucine + lecithin, the maximum % of CO_2_ was 8.01 at 1 min in the top layer of the GH block, while in the middle and bottom layers the maximum % of CO_2_ was 3.22 (1 min) and 1.23 (1 min) respectively. Overall, the top layer captured more CO_2_, followed by the middle and bottom layers. In case of methionine + lecithin, the maximum % of CO_2_ was 8.01 at 1 min in the top layer of the GH block, while in the middle and bottom layers the maximum % of CO_2_ was 3.22 (1 min) and 1.23 (1 min) respectively. Overall, the top layer captured more CO_2_, followed by the middle and bottom layers. In case of methionine + leucine + lecithin, the maximum % of CO_2_ was 9.92 at 1 min in the top layer of the GH block, while in the bottom and middle layers the maximum % of CO_2_ was 8.02 (1 min) and 7.84 (1 min) respectively. Overall, the top layer captured more CO_2_, followed by the bottom and middle layers. Therefore, from the graphs, the top performed the better followed by the middle, and then the bottom for the amino acids cysteine, methionine, leucine + methionine, leucine + lecithin, methionine + lecithin, and methionine + leucine + lecithin, while for the amino acids leucine and valine, the middle one was better at certain times, followed by the top and bottom. The reason can be explained via CO_2_ GH nucleation process that occurs at the liquid–gas interface near the reactor wall, where the temperature is the lowest; next, CO_2_ GH crystals develop as a porous structure on the reactor wall. To encourage continuous CO_2_ generation, the solution diffuses from the bulk phase to the porous structure due to the capillary forces^1,60,61^. Thus, the rapid GH development was seen in both the upward and downward directions, i.e., in the overlaying gas phase and the underlying bulk solution, following GH nucleation at the gas–liquid interface. Therefore, it was logical that nucleation occurred across the entire gas–liquid interface rather than just at the three-phase interface points in this case, and that hydrate growth propagated vertically along the length of the reactor (downward and upward directions) rather than from the three-phase interface points toward the reactor's centre^21,41,42,62^. Another reason could be the handling of the GH, as for the experimental setup, it takes around 10 min.

## Discussion

GH production is hampered by difficulties such as the high pressures necessary for GH creation, sluggish gas uptake rates, and low final gas uptakes realized. Amino acids are powerful kinetic GH promoters that operate in a clean manner (no foam generation) and stand out as excellent alternatives to chemical additives for CO_2_ GH based technological applications especially for food applications. However, further research is needed to understand which amino acids might be effective kinetic promoters for various GHs. More study is also required to understand the many methods by which amino acids increase GH production. Only two amino acids have shown the appropriate kinetic promoting activity for CO_2_ GH production in the current study are methionine and leucine, both hydrophobic molecules. The most likely way for amino acids to kinetically increase GH generation is via changing the shape of the GH being generated. However, certain literature studies dispute this observation, prompting more research. As it was evident from the study, the GH is more susceptible to dissociation at high temperatures than at lower temperatures. Hence, the stability of the GH decreases with an increase in temperature. But the addition of promoters, especially leucine + methionine + lecithin really increased the CO_2_ uptake during the GH formation. Therefore, it is imaginable that surfactants and hydrophobic amino acids would influence CO_2_ GH formation either by reducing the time required for hydrate nucleation and/or increasing the rate of GH development^2,35,39,53,59^. Also, some serious efforts are required to bridge the gap between the applications of CO_2_ GH as a leavening agent from a food industrial perspective.

## Methods

### Materials: amino acids and surfactant

Three essential amino acids (need to be acquired from outside the body for the human body to function) leucine, valine, and methionine, and one nonessential (produced naturally in the body) amino acid , cysteine were used as kinetic promoter for CO_2_ GH production^60^. Selection of the amino acids as kinetic promoters and lecithin as a surfactant was done on the basis of previous findings reported by Cai et al^1^, Prasad and Kiran^16^, Nasir et al.^61^, and Wang et al.^59^. All the amino acids and lecithin were purchased from the Sigma Aldrich Company with ≥ 98% purity.

### Detailed description of the GH reactor

**Figure 6:**
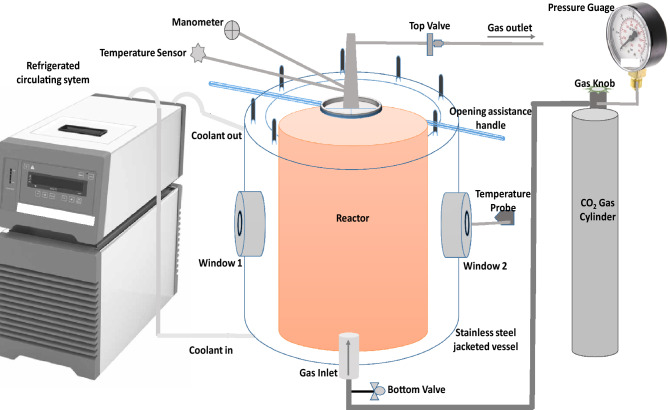
Schematic representation of gas hydrate reactor assembly.

The GH reactor was installed at the Department of Process Analytics and Cereal Science, University of Hohenheim, Stuttgart, Germany in collaboration with the installation assistance from the Institute of Fluid Mechanics (LSTME), FAU Erlangen-Nuremberg, Erlangen, Germany. The reactor installed was borrowed from FAU, Erlangen, Germany for the project^63^. The schematic GH reactor assembly is shown in Fig. 6. The reactor was fitted with two circular type viewing windows (at the front and back) to allow visual inspection of the reactor contents during GH formation/dissociation. The pressure chamber in the reactor can withstand high-pressure conditions of 4.5 MPa due to its stainless-steel body. The length of GH reactor chamber was 29.5 cm. The 1500 mL volume reactor was fitted with a constantly refrigerated circulator temperature bath (IKA RC 2 Green basic model, IKA-Werke GmbH & Co. KG, Germany) in which cooling was achieved by the circulation of propane as coolant. The pump speed of the refrigerated circulator temperature bath was set to 3000 rpm. The coolant flowing from a chilled bath was capable of keeping the bath temperature within ± 0.1 °C of the set point. The chilled water circulator provided cold thermal energy to keep the reaction chamber at the experimental temperature. The pressure regulator was in charge of regulating the starting pressure (WIKA type 111.10 model, Landelfeld GmbH, Germany) with a pressure range of 0 to 60 bars, and a temperature range of − 20 °C to 60 °C in a gas cylinder. The pressure in the reactor vessel was measured with a WIKA pressure transducer (WIKA, EN 837–1 for pressure range of 0–45 bars). The uncertainty of the pressure measurements was ± 1 bar. The experimental temperature was measured using two thermometers, whose accuracy was ± 0.01 °C. One thermometer was situated at the window side of the reactor vessel, while the other extended at the top of the reactor vessel (Fig. 6). Also, to aid taking out the CO_2_ GH out of the reactor, a cylindrical hollow piston made of stainless steel was fabricated additionally, and placed inside the reactor during the formation of GH.

### CO_2_ GH formation in the reactor

The reactor vessel was washed thoroughly by water twice with the help of an aspirator pump before the start of the experiment so as to ensure no solute contamination. The operation of the GH reactor was done by first closing the valve on the bottom. The reactor was opened with the help of a fork lift and was filled with distilled cold water (500 mL). Thereafter, the reactor was closed after wiping off any trace of water or dust for proper closure and pressure build up. Then the upper valve was closed, which was used as an air outlet. Afterwards, the temperature inside the reactor was checked using the thermo-couple, and the reactor was kept in an ideal position until the temperature inside was low, around 0.3–1 °C. A check over the pressure build-up and leakage during the waiting period was performed. CO_2_ gas hydrates were produced by the addition of 500 mL of distilled water into the reactor vessel. The lid of the reactor vessel was closed, and the CO_2_ gas was directed inside the reactor through its lower valve. Once the optimum pressure (32–37 bars) and lower temperatures (0–1 °C) were reached inside the reactor, the upper valve located on the lid was opened slightly so that a constant bubbling of CO_2_ gas in the water could take place, leading to the dissolution of CO_2_ gas. Thereafter, the CO_2_ GH production was initiated with the nucleation reaction inside the reactor, which came to an end within 3–4 h. To produce the CO_2_ GH with promoters, the aforesaid process was followed along with the addition of promoters to the distilled water. Although many promoters are available for enhancing CO_2_ GH production, still based on the literature survey, only food grade promoters were selected for the study as the CO_2_ GH produced with the application of promoters would ultimately be used in the baking processes in the next research applications of the produced CO_2_ GH. Therefore, four food grade kinetic promoters and one surfactant were studied, namely leucine, methionine, cysteine, valine, and lecithin, respectively. Each kinetic amino acid promoters/surfactant sample was weighed using an electronic balance with an error of ± 0.1 mg. In all of the trials, distilled water was utilized. The effects of these promoters and surfactant were investigated individually or in combinations. The four amino acids were added in an amount of 0.5 g (% wt), and surfactant lecithin in an amount of 5 g (1% of water) was utilized irrespective of being used individually or in combinations. Also, each experiment was conducted at least three times to check the reproducibility of results. A dramatic pressure decrease at a specific temperature signalled the development of hydrates. An insignificant decline in head-pressure in the reactor over a prolonged period of time suggested saturation in hydrate conversion.

### Determination of the amount of CO_2_ gas release from the GH

**Figure 7:**
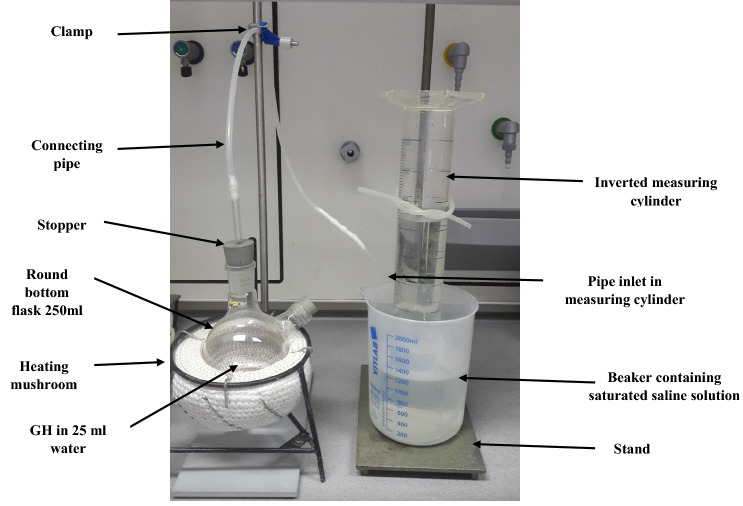
Experimental set up for CO_2_ gas release measurement from the GH at 90 °C.

The experimental setup for CO_2_ gas release measurement from the GH at 90 °C is shown in Fig. 7. A 2000 ml measuring cylinder was placed in an upright position in a 2000 ml beaker containing saturated saline solution without air bubbles. The GH (7.5 -10 g) was mixed with 25 ml of water in a 250 ml round bottom flask and screwed in tightly (air-tight) with a connecting pipe running towards the inverted measuring cylinder. Then the same connecting pipe was placed inside the upright measuring cylinder without air bubbles to see the production of the carbon dioxide bubbles. The reading for the decrease in volume inside the inverted measuring cylinder was calculated for the amount of carbon dioxide released every minute. The reaction was ended (approx. 20 min) and the percentage of carbon dioxide was calculated by Eq. (1):

1 $$ {\text{Calculation}}\;{\text{of}}\;{\text{CO}}_{{2}} \left( \% \right) = \left( {0.{1963}\;{\text{V}}} \right)/{\text{E}} $$

where *V* is the volume of the saline solution that has flown out in ml, *E* is the weight of GH in grams, and 1.963 is the liter weight of CO_2_ in grams as per the ideal gas equation.

The dissociation experiments at high temperature was performed on the same day when the CO_2_ GH was produced so as to strike out the effect of storage of the produced GH. The dissociation of CO_2_ GH with amino acid kinetic promoters and without promoters (normal water GH) was studied at a high temperature of 90 °C for a period of 20 min to understand the percentage of CO_2_ gas content in each batch, and to select the best promoter that aids CO_2_ gas entrapment along with stability at a high temperature for use in the bakery products.

### Human or animal rights

This article does not contain any studies with human participants or animals performed by any of the authors.

## Data Availability

All data generated or analyzed during this study are included in this published article.
